# Prevalence of Oral Human Papillomavirus Infection Among Australian Indigenous Adults

**DOI:** 10.1001/jamanetworkopen.2020.4951

**Published:** 2020-06-08

**Authors:** Lisa M. Jamieson, Annika Antonsson, Gail Garvey, Xiangqun Ju, Megan Smith, Richard M. Logan, Newell W. Johnson, Joanne Hedges, Sneha Sethi, Terry Dunbar, Cathy Leane, Isaac Hill, Alex Brown, David Roder, Marjorie De Souza, Karen Canfell

**Affiliations:** 1 Australian Research Centre for Population Oral Health, University of Adelaide, Adelaide, Australia; 2 QIMR Berghofer Medical Research Institute, Royal Brisbane Hospital, Brisbane, Australia; 3 Menzies School of Health Research, Spring Hill, Australia; 4 Cancer Council New South Wales, Sydney, Australia; 5 Sydney Medical School, University of Sydney, Sydney, Australia; 6 Adelaide Dental School, University of Adelaide, Australia; 7 Menzies Health Institute Queensland, Griffith University and Faculty of Dentistry, Queensland, Australia; 8 Oral and Craniofacial Sciences, King’s College, London, United Kingdom; 9 National Centre for Epidemiology and Population Health, ANU College of Health and Medicine, Australian National University, Australian Capital Territory, Australia; 10 Strategic Partnerships, Aboriginal Health Division Women's and Children's Health Network, Adelaide, Adelaide, Australia; 11 Aboriginal Health Council of South Australia, Adelaide, Australia; 12 Wardliparingga Aboriginal Research Unit, South Australian Health and Medical Research Institute, Adelaide, Australia; 13 University of South Australia School of Health Sciences, Adelaide, Australia

## Abstract

**Importance:**

Human papillomavirus (HPV) infection is associated with oropharyngeal squamous cell carcinoma. International estimates suggest overall oral HPV prevalence is 7.5%, with prevalence of oral HPV types 16 and 18 being 1.6%; prior Australian estimates suggest oral HPV prevalence is 2.3%, with HPV-16 and HPV-18 being 1.3%.

**Objectives:**

To estimate the prevalence of oral HPV infection among Indigenous Australians and to report the prevalence of factors associated with high-risk HPV types (ie, HPV-16 and HPV-18) and HPV types linked with Heck disease (ie, HPV-13 and HPV-32).

**Design, Setting, and Participants:**

This cross-sectional study analyzed HPV screening results from saliva samples collected from 1011 Indigenous Australians between February 2018 and January 2019. Data were analyzed from May 2018 to May 2019. Recruitment occurred through Aboriginal Community Controlled Health Organisations in South Australia. Eligibility included identifying as Indigenous, residing in South Australia, and being aged 18 years or older.

**Main Outcomes and Measures:**

Saliva samples were collected, with microbial DNA for genotyping extracted. Sociodemographic parameters, health-related behaviors, and sexual history data were collected. Analyses were stratified by sex as well as by HPV types 13 and 32 (Heck disease) and 16 and 18 (high risk of oropharyngeal squamous cell carcinoma). Multivariable analyses were conducted to obtain adjusted odds ratios (ORs).

**Results:**

Data were obtained for 910 participants (median [interquartile range] age, 37 [27-51] years); 595 participants (65%) were female and 572 (63%) resided in nonmetropolitan locations. In all, 321 saliva samples (35.3%; 95% CI, 32.2%-38.4%) were positive for oral HPV (106 [33.7%] men; 215 [36.1%] women). The highest prevalence was found for HPV types 13 and 32 (207 [22.7%] total; 60 [19.0%] men; 147 [24.7%] women) followed by HPV types 16 and 18 (30 [3.3%] total; 9 [2.9%] men; 21 [3.5%] women). After multivariable analysis, risk factors associated with HPV types 13 and 32 included nonmetropolitan residential status (OR, 2.06; 95% CI, 1.10-3.88) and not having had a tonsillectomy (OR, 2.74; 95% CI, 1.05-7.16). Among women, having obtained a high school education or less was associated with lower odds of HPV-16 and HPV-18 infection (OR, 0.16; 95% CI, 0.03-0.97).

**Conclusions and Relevance:**

Prevalence of oral HPV infection in a large sample of Indigenous Australians was high, with one-third testing positive. The most prevalent HPV types were those associated with Heck disease. The prevalence of HPV types associated with oropharyngeal squamous cell carcinoma exceeded both Australian and international population-level estimates.

## Key Points

**Question:**

What is the prevalence of oral human papillomavirus (HPV) infection among Indigenous Australians, a group at risk of oropharyngeal squamous cell carcinoma?

**Findings:**

This cross-sectional study examined 910 Indigenous Australians for HPV infection with a particular focus on high-risk HPV types. Thirty-five percent of study participants had an oral HPV infection, 15 times the incidence reported in a study of young Australians and 5 times that reported in a systematic review from other countries.

**Meaning:**

The findings of this study indicate that Indigenous Australians may be at higher risk of developing HPV-related oral cancer, which suggests that increased HPV vaccination coverage among this vulnerable population may be beneficial.

## Teaser

This cross-sectional study of 910 Indigenous Australian adults examines incidence of and demographic patterns in oral human papillomavirus (HPV) infection.

## Introduction

Oropharyngeal squamous cell carcinoma (OPSCC) associated with human papillomavirus (HPV) disproportionately affects men and has one of the most rapidly increasing incidences of any cancer in high-income countries.^1^ The increased incidence is particularly noted among younger cohorts with minimal exposure to smoking and alcohol, the risk factors most commonly associated with OPSCC; the increased HPV incidence may be attributable to oral exposure to infected anogenital sites with changing sexual behaviors.^2,3^ Globally, the proportion of OPSCC attributable to HPV has been estimated as 23% to 31%; however, this varies by setting, particularly regarding exposure to HPV, tobacco, and alcohol.^4,5^ In Australia, Hong et al^3^ reported a more than 3-fold increase in the percentage of HPV-positive OPSCC in the last 2 decades, from 20% to 63%; HPV-16 is the most common type in HPV-positive OPSCC, although HPV-18 also plays a role.^6^ The 2 types together account for 85% of HPV-positive OPSCC (83% HPV-16; 2% HPV-18).^5^ In 2012, the incidence of OPSCC in men overtook that of cervical cancer in women in the United States,^7^ and similar findings were observed in the United Kingdom in 2016.^8^ Australian OPSCC incidence trends are in line with other high-income countries.^3^ While these countries have also experienced reduced rates of cervical cancer due to successful screening initiatives, the increase in OPSCC remains notable.

Focal epithelial hyperplasia, or Heck disease, is a relatively benign and rare condition caused by oral HPV types 13 or 32.^9,10,11,12^ It was first identified among a Navajo population in the United States^13^ and has since been reported among other indigenous groups throughout the world.^14,15^ Heck disease is characterized by multiple white or pink papules that occur diffusely throughout the oral cavity, with morphology that can present as slightly pale, smooth, or roughened surfaces. Although the papules will spontaneously regress without treatment over time, some patients opt for excisional biopsy for functional or cosmetic purposes.^16^

Aboriginal and Torres Strait Islanders (hereafter respectfully termed *Indigenous*) are the first peoples of Australia, having resided in the country for more than 50 000 years. Contemporary Indigenous Australians represent 3.3% of the total Australian population.^17^ They are overrepresented in almost all cancer statistics, including cancers of the head and neck.^18^ In an analysis of cancer registry data, Banham et al^19^ reported that in comparison with the general population of Australia, Indigenous individuals were 10 years younger at diagnosis, to be residents of geographically remote locations, and to have primary cancer sites of the head and neck, lung, liver, and cervix. In 2009 to 2013, Indigenous Australians were 1.9 times more likely to be diagnosed with head and neck cancer than non–Indigenous Australians, and were 3.4 times more likely to die.^20^ Risk of cancer death was associated with advanced stage at first observation, with more Indigenous than non-Indigenous individuals having distant metastases at diagnosis. Although HPV vaccine coverage across Indigenous adolescents in Australia is high, course completion is generally lower.^21^ Evidence suggests that while HPV infection in other anatomical sites is similar to non–Indigenous Australians, Indigenous Australians experience a higher prevalence of risk factors and other HPV genotypes.^22^

In a systematic review of 9 studies that collected oral HPV data from 3762 cancer-free, HIV-negative individuals from the United States, Brazil, Mexico, and Finland, Wood et al^23^ reported that 7.5 % (95% CI, 6.7%-8.4%) had an oral infection with any HPV type at baseline. In a study involving 307 Australian university students (aged 18-35 years), 7 students (6 men and 1 woman; 2.3%; 95% CI, 0.6%-3.9%) tested positive for oral HPV infection. Those positive for oral HPV were more likely to have received oral sex from more partners in their lifetime.^24^

Given the high risk of Indigenous Australians having both oral HPV infection and OPSCC and the potential benefits of HPV vaccination, the aims of this study were as follows: (1) to estimate the prevalence of oral HPV infection among Indigenous Australians; (2) to identify risk factors associated with Heck disease HPV types (HPV-13 and HPV-32); and (3) to identify risk factors associated with OPSCC-related HPV types (HPV-16 and HPV-18). We hypothesized that levels of any oral HPV infection, oral HPV infection associated with Heck disease (HPV-13 and HPV-32), and oral HPV infection associated with OPSCC (HPV-16 and HPV-18) among an Indigenous adult population would be higher than overall population estimates. We additionally hypothesized that risk factors for oral HPV infection would include male sex, social disadvantage, tobacco use, and early and frequent sexual activity.

## Methods

### Study Design and Participants

We used a large convenience sample (n = 1011) of adults aged 18 years or older who identified as being Indigenous in the Australian state of South Australia. Data were collected between February 2018 and January 2019 as part of a broader study investigating HPV and OPSCC among Indigenous Australians^25^ and were analyzed from October 2018 to July 2019. The study was governed by an Indigenous Reference Group, with data collected by trained Indigenous research officers. Participants were primarily recruited through Aboriginal Community Controlled Health Organisations, which were key stakeholders in the study. After having the study explained and signing informed consent forms, participants were asked to complete a questionnaire (with assistance from study staff if requested) that contained information on sociodemographic characteristics, health-related behaviors including tobacco and alcohol use, and sexual history. Participants then provided a saliva sample through spitting and dribbling that was collected in a commercially available kit (DNA Genotek Inc), from which microbial DNA was extracted for genotyping.

Ethical approval was received from the University of Adelaide Human Research Ethics Committee and the Aboriginal Health Council of South Australia’s Human Research Ethics Committee. This study follows the Strengthening the Reporting of Observational Studies in Epidemiology (STROBE) reporting guideline for cross-sectional studies.

### Self-reported Data

Sociodemographic characteristics included age, sex, geographic location, education, income, ownership of a means-tested government health care card, number of people in house the previous night, and car ownership. Health-related behaviors surveyed included tobacco, alcohol, nonprescription tobacco substitute (ie, vaporizer or e-cigarette), recreational drug use, having ever been diagnosed with an HPV infection (self-reported), HPV vaccination status (self-reported) and having had a tonsillectomy. Sexual behaviors across lifetime included number of people passionately kissed, having ever given oral sex, having ever received oral sex, having experienced sexual intercourse, and current relationship status. Age was dichotomized based on a median split. Geographic location was defined as metropolitan (ie, residing in Adelaide, South Australia’s capital city) and nonmetropolitan (residing elsewhere in the state). Highest educational attainment was categorized as high school or less and trade, TAFE, or university (TAFE stands for technical and further education and provides training for vocational occupations). Income was defined as job or Centrelink or other (Centrelink is the government agency responsible for means-tested welfare payments). Similarly, enrollment in government-administered health care card is means tested, and enables access to services such as publicly funded dental care. Number of people in house the previous night was used as a proxy measure of socioeconomic status (as used in studies conducted by the Australian Institute of Health and Welfare^26^) and was categorized according to whether the total number of people spending the previous night in the participant’s residence was 4 or more or less than 4. Tobacco smoking status was defined as currently smoking tobacco, formerly smoked, and never smoked, while response categories for alcohol consumption included daily, weekly, monthly, or never.

### Laboratory Analysis

A viral kit for DNA extraction (Promega Maxwell) was used. β-globin polymerase chain reaction (PCR) with the primers PCO3 and PCO4 was carried out on all samples to ensure the presence of human DNA and that no PCR-inhibiting agents were present.^27^ All samples were analyzed with a nested PCR system (MY09/11) and GP^5+/6+^ that detects most mucosal HPV types and all high-risk HPV types that have oncogenic potential in mucosal tissue.^28,29^ All HPV-positive DNA samples were sequenced to confirm viral DNA sequences. For sequencing, HPV-positive PCR products were purified with a PCR purification kit in a magnetic 96-ring SPRIplate (Agencourt Biosciences). Sequencing reactions were performed containing the purified PCR products together with GP^+^ primer and BigDye Terminator. Sequence reactions were purified with a dye-terminator removal kit (Agencourt Biosciences) in a magnetic 96-ring SPRIplate. Direct sequencing was conducted, and sequence reactions were analyzed with an automated DNA sequencer (Applied Biosystems). The DNA sequences were compared with available sequences in GenBank through the National Center for Biotechnology Information BLASTn suite server.^30^ Participants with β-globin–positive saliva samples were included in the data analysis. (β-globin is a DNA integrity check; any samples with negative β-globin were invalid.)

### Statistical Analysis

Basic descriptive analyses were conducted to ascertain frequencies of all HPV types detected and associations with sociodemographic data, health-related behaviors, and sexual history characteristics. Bivariate and multivariable logistic regression analyses were then conducted to identify risk factors for infection with HPV types associated with OPSCC (HPV-16 and HPV-18) and with Heck disease (HPV-13 and HPV-32). All data were stratified by sex. Differences were denoted to be statistically significant when 95% CIs did not overlap, and the χ^2^ *P* value in 2-tailed tests was less than .05. Odds ratios (ORs) with 95% CIs were calculated in the multivariable analyses. SAS version 9.4 (SAS Institute) was used for all analyses.

## Results

A total of 910 β-globin–positive saliva samples were collected from Indigenous residents of South Australia aged 18 years or older. Among participants, the median (interquartile range [IQR]) age was 37 (27-51) years, with a median (IQR) age of 37 (27-49) years for men and 38 (27-52) years for women. More than half (51.6%; 95% CI, 48.4%-54.9%) of participants were aged 37 years or older (Table 1). Two-thirds (65.4%; 95% CI, 62.3%-68.5%) were women and 63% resided in nonmetropolitan locations (63.0%; 95% CI, 59.8%-66.1%). Overall, 68.0% (95% CI, 65.0%-71.1%) of participants reported a highest level of eductional attainment as high school or less, and more than three-quarters (76.0%; 95% CI, 73.2%-78.8%) received their income through Centrelink. More than one-third (36.7%; 95% CI, 33.5%-40.0%) of participants had 4 or more people in their house the previous night. Fifty-five percent (55.2%; 95% CI, 52.0%-58.5%) of participants owned their own car. Nearly 60% (58.2%; 95% CI, 54.9%-61.5%) currently smoked tobacco, and approximately 12% (11.6%; 95% CI, 9.5%-13.8%) reported currently smoking nonprescription tobacco substitutes. Nearly one-quarter (24.1%; 95% CI, 21.2%-26.9%) of participants consumed alcohol on a weekly basis, while approximately 21.1% (95% CI, 18.4%-23.7%) reported currently using recreational drugs. Two percent (2.0%; 95% CI, 1.1%-2.9%) of participants reported having ever had an HPV infection, but 17.1% (95% CI, 14.6%-19.6%) did not know. Approximately 8% (8.3%; 95% CI, 6.5%-10.1%) had been vaccinated against HPV (bearing in mind most participants would not have been eligible for free public HPV vaccination), although 34.1% (95% CI, 31.0%-37.2%) did not know their vaccination status. Approximately 13% (12.9%; 95% CI, 10.7%-15.1%) reported having had a tonsillectomy. Nearly two-thirds (65.4%; 95% CI, 62.1%-68.6%) of participants reported having passionately kissed 4 or more people, 64.6% (95% CI, 61.3%-67.9%) had given oral sex, and 64.7% (95% CI, 61.4%-68.0%) had received oral sex. Almost all (94.8%; 95% CI, 93.2%-96.3%) participants reported having had sexual intercourse, with 40% commencing sexual activity before the age of 16 years, with 64.0% of participants (95% CI, 60.5%-67.4%) reporting 4 or more sexual partners over their lifetime. Nearly all (93.6%; 95% CI, 92.0%-95.3%) participants reported sexual encounters with people predominantly of the opposite sex. More than half (50.9%; 95% CI, 47.4%-54.3%) were in current, stable long-term relationships.

**Table 1.** Sociodemographic Characteristics, Health-Related Behaviors, and Sexual History, Stratified by Sex

Characteristic	HPV-OPC study, % (95% CI)		
	All (N = 910)	Men (n = 315)	Women (n = 595)
Total	100	34.6 (31.5-37.7)	65.4 (62.3-68.5)
Age, y			
≥37	51.6 (48.4-54.9)	51.7 (46.2-57.3)	51.6 (47.6-55.6)
<37	48.4 (45.1-51.6)	48.3 (42.7-53.8)	48.4 (44.4-52.4)
Geographic location			
Nonmetropolitan	63.0 (59.8-66.1)	60.8 (55.4-66.3)	64.1 (60.3-68.0)
Metropolitan	37.0 (33.9-40.2)	39.2 (33.7-44.6)	35.9 (32.0-39.8)
Level of education			
High school	68.0 (65.0-71.1)	60.8 (55.4-66.3)	66.7 (62.8-70.5)
Trade/TAFE/university^a^	32.0 (28.9-35.0)	39.2 (33.7-44.6)	33.3 (29.5-37.2)
Income^b^			
Centrelink or other	76.0 (73.2-78.8)	69.2 (64.1-74.4)	79.6 (76.3-82.8)
Job	24.0 (21.2-26.8)	30.8 (25.6-35.9)^d^	20.4 (17.2-23.8)
Health care card ownership^c^			
Yes	78.8 (76.1-81.5)	76.7 (71.8-81.5)	79.9 (76.6-83.2)
No	21.2 (18.5-23.9)	23.3 (18.5-28.2)	20.1 (16.8-23.4)
People in house previous night, No.			
≥4	36.7 (33.5-40.0)	34.5 (28.9-40.1)	37.9 (33.8-42.0)
<4	63.3 (60.0-66.5)	65.5 (59.9-71.1)	62.1 (58.0-66.2)
Own car			
No	44.8 (41.5-48.0)	45.2 (39.6-50.7)	44.6 (40.6-48.6)
Yes	55.2 (52.0-58.5)	54.8 (49.3-60.4)	55.4 (51.4-59.4)
Tobacco smoking status			
Current	58.2 (54.9-61.5)	65.9 (60.5-71.3)^d^	54.0 (49.9-58.2)
Former	12.4 (10.2-14.6)	9.6 (6.3-12.9)	14.0 (11.0-16.8)
Never	29.4 (26.3-32.4)	24.5 (19.6-29.4)	32.0 (28.1-35.9)
Alcohol consumption			
Daily	3.6 (2.4-4.8)	5.2 (2.7-7.7)	2.8 (1.4-4.1)
Weekly	24.1 (21.2-26.9)	35.9 (30.5-41.4)^d^	17.8 (14.7-20.9)
Monthly	37.1 (33.9-40.3)	32.7 (27.4-38.0)	39.4 (35.4-43.4)
Never	35.3 (32.1-38.4)	26.1 (21.2-31.1)^d^	40.1 (36.1-44.1)
Use of nonprescription tobacco substitutes, eg, vaporizer or e-cigarette			
Current	11.6 (9.5-13.8)	13.4 (9.5-17.3)	10.7 (8.1-13.2)
Former	19.1 (16.4-21.7)	22.8 (18.0-27.6)	17.1 (14.0-20.2)
Never	69.3 (66.2-72.4)	63.8 (58.3-69.2)	72.2 (68.5-76.0)
Use of recreational drugs			
Current	21.1 (18.4-23.7)	27.8 (22.9-32.9)^d^	17.4 (14.4-20.5)
Former	33.7 (30.6-36.8)	41.3 (35.9-46.8)	29.6 (25.9-33.3)
Never	45.3 (42.0-48.5)	30.8 (25.6-35.9)	53.0 (48.9-57.0)
Ever diagnosed with HPV			
Yes	2.0 (1.1-2.9)	0.3 (0.0-1.0)^d^	2.9 (1.5-4.2)
No	80.9 (78.3-83.5)	81.7 (77.3-86.0)	80.5 (77.3-83.7)
Don’t know	17.1 (14.6-19.6)	18.0 (13.7-22.3)	16.6 (13.6-19.6)
Ever received HPV vaccination			
Yes	8.3 (6.5-10.1)	2.2 (0.6-3.9)^d^	11.5 (8.9-14.1)
No	57.5 (54.3-60.8)	63.1 (57.8-68.5)	54.6 (50.5-58.6)
Don’t know	34.1 (31.0-37.2)	34.6 (29.3-39.9)	33.9 (30.1-37.7)
Tonsils removed			
Yes	12.9 (10.7-15.1)	9.9 (6.5-13.3)	14.5 (11.6-17.4)
No	81.9 (79.3-84.4)	80.9 (76.4-85.3)	82.4 (79.3-85.5)
Don’t know	5.2 (3.7-6.8)	9.2 (6.5-13.3)	3.1 (1.7-4.5)
In life, how many passionately kissed			
≥4	65.4 (62.1-68.6)	69.5 (64.1-75.0)	63.2 (29.1-67.3)
<4	34.6 (31.4-37.9)	30.5 (25.0-35.9)	36.8 (32.7-40.9)
Ever given oral sex			
Yes	64.6 (61.3-67.9)	63.0 (57.3-68.7)	65.4 (61.4-69.5)
No	35.4 (32.1-38.7)	37.0 (31.3-42.7)	34.6 (30.5-38.6)
If yes, age when first gave oral sex, y			
<16	24.6 (20.8-28.3)	28.6 (21.8-35.3)	22.5 (18.1-27.0)
≥16	75.4 (71.7-79.2)	71.4 (64.7-78.2)	77.5 (73.0-81.9)
People given oral sex to in lifetime, No.			
>3	44.3 (40.0-48.6)	54.6 (47.1-62.1)^d^	39.1 (34.0-44.3)
≤3	55.7 (51.4-60.0)	45.4 (37.9-52.9)	60.9 (55.7-66.0)
Ever received oral sex			
Yes	64.7 (61.4-68.0)	68.0 (62.5-73.5)	62.9 (58.8-67.1)
No	35.3 (32.0-38.6)	32.0 (26.5-37.5)	37.1 (32.9-41.2)
If yes, age when first received oral sex, y			
<16	28.4 (24.5-32.3)	42.0 (34.9-49.1)^d^	20.4 (16.0-24.9)
≥16	71.6 (67.7-75.5)	58.0 (50.9-65.1)	79.6 (75.1-84.0)
People received oral sex from in lifetime, No.			
>3	49.9 (45.6-54.2)	68.6 (61.9-75.3)^d^	39.1 (33.7-44.4)
≤3	50.1 (45.8-54.4)	31.4 (24.7-38.1)	60.9 (55.6-66.3)
Sexual intercourse with another person			
Yes	94.8 (93.2-96.3)	95.3 (92.7-97.8)	94.5 (92.6-96.5)
No	5.2 (3.7-6.8)	4.7 (2.2-7.3)	5.5 (3.5-7.4)
If yes, age when first had sex, y			
<16	40.0 (36.5-43.5)	47.3 (41.2-53.4)^d^	36.2 (31.9-40.4)
≥16	60.0 (56.5-63.5)	52.7 (46.6-58.8)	63.8 (59.6-68.1)
Sexual partners, No.			
≥4	64.0 (60.5-67.4)	75.1 (69.8-80.4)^d^	58.1 (53.8-62.5)
<4	36.0 (32.6-39.5)	24.9 (19.6-30.2)	41.9 (37.5-46.2)
In lifetime, sexual encounters have been mostly			
Heterosexual	93.6 (92.0-95.3)	95.6 (93.2-98.1)	92.6 (90.4-94.9)
Homosexual	0.7 (0.2-1.3)	1.5 (0.0-2.9)	0.4 (0.0-0.9)
Bisexual	5.6 (4.0-7.2)	2.9 (0.9-4.9)^d^	7.0 (4.8-9.2)
Current relationship status			
Stable long-term	50.9 (47.4-54.3)	49.6 (43.7-55.6)	51.5 (47.2-55.7)
Short-term	5.8 (4.2-7.4)	4.0 (1.7-6.3)	6.7 (4.6-8.8)
Single	43.3 (39.9-46.8)	46.4 (40.5-52.3)	41.8 (37.6-46.0)

Abbreviations: HPV, human papillomavirus; HPV-OPC, human papillomavirus-associated oropharynx cancer; TAFE, technical and further education.

^a^ TAFE provides training for vocational occupations.

^b^ Centrelink is the government agency responsible for welfare payments to those means-tested to be eligible.

^c^ Ownership of a government-administered health care card is means-tested and enables access to services such as publicly funded dental care.

^d^ Difference statistically significant as denoted by nonoverlapping 95% CIs; *P* < .05.

More than one-third (35.3%) were positive for an oral HPV infection (Table 2); 33.7% of men and 36.1% of women. By far the most prevalent HPV types were those associated with Heck disease (HPV-13 and HPV-32) (22.8%; 19.1% in men and 24.7% in women). The next most prevalent HPV types were those associated with OPSCC (HPV-16 and HPV-18) (3.3%; 2.9% in men and 3.5% in women). A total of 38 HPV types were found, with the number of participants per type ranging from 1 to 119. There were no participants with multiple oral HPV types.

**Table 2.** Oral HPV Types Among 910 Indigenous Adults in South Australia

Characteristic	Participants, No. (%)		
	Total	Men	Women
Total	910 (100.0)	315 (100.0)	595 (100.0)
Positive ≥1 oral HPV type	321 (35.3)	106 (33.7)	215 (36.1)
Positive oral			
HPV-13 and/or HPV-32	207 (22.8)	60 (19.1)	147 (24.7)
HPV-16 and/or HPV-18	30 (3.3)	9 (2.9)	21 (3.5)
HPV type			
3	2 (0.2)	0	2 (0.3)
6	3 (0.3)	2 (0.6)	1 (0.2)
7	1 (0.1)	1 (0.3)	0
10	1 (0.1)	0	1 (0.2)
13	88 (9.7)	27 (8.6)	61 (10.3)
16	14 (1.5)	4 (1.3)	10 (1.7)
18	16 (1.8)	5 (1.6)	11 (1.9)
30	2 (0.2)	2 (0.6)	0
31	1 (0.1)	1 (0.3)	0
32	119 (13.1)	33 (10.5)	86 (14.5)
33	1 (0.1)	0	1 (0.2)
34	1 (0.1)	1 (0.3)	0
35	3 (0.3)	2 (0.6)	1 (0.2)
39	2 (0.2)	2 (0.6)	0
40	1 (0.1)	1 (0.3)	0
42	1 (0.1)	1 (0.3)	0
44	1 (0.1)	1 (0.3)	0
45	3 (0.3)	3 (1.0)	0
51	1 (0.1)	0	1 (0.2)
52	2 (0.2)	0	2 (0.3)
53	2 (0.2)	1 (0.3)	1 (0.2)
54	1 (0.1)	0	1 (0.2)
56	4 (0.4)	2 (0.6)	2 (0.3)
58	5 (0.6)	3 (1.0)	2 (0.3)
59	5 (0.6)	3 (1.0)	2 (0.3)
62	1 (0.1)	0	1 (0.2)
66	9 (1.0)	3 (1.0)	6 (1.0)
67	2 (0.2)	1 (0.3)	1 (0.2)
68	1 (0.1)	0	1 (0.2)
69	8 (0.9)	3 (1.0)	5 (0.8)
72	7 (0.8)	1 (0.3)	6 (1.0)
73	1 (0.1)	0	1 (0.2)
81	3 (0.3)	1 (0.3)	2 (0.3)
82	1 (0.1)	0	1 (0.2)
84	1 (0.1)	0	1 (0.2)
87	1 (0.1)	1 (0.3)	0
90	5 (0.6)	1 (0.3)	4 (0.7)
106	1 (0.1)	0	1 (0.2)

Abbreviation: HPV, human papillomavirus.

In bivariate analysis, we found an association between having any oral HPV type (Table 3) and receiving income through Centrelink compared with a job (prevalence, 37.5% [95% CI, 34.8%-41.1%] vs 28.7% [95% CI, 22.7%-34.7%]). In bivariate analysis, we found an association between having an HPV type associated with Heck disease and residing in a nonmetropolitan location compared with a metropolitan location (prevalence, 28.5% [95% CI, 24.8%-32.3%] vs 13.1% [9.5%-16.7%]), not owning a car compared with owning a car (prevalence, 28.0% [95% CI, 23.6%-32.4%] vs 18.5% [95% CI, 15.1%-21.9%]), having not had a tonsillectomy compared with having had a tonsillectomy (prevalence, 24.1% [95% CI, 20.9%-27.2%] vs 14.0% [95% CI, 7.6%-20.4%]), having never given oral sex compared with having given oral sex (prevalence, 27% [95% CI, 22.3%-32.6%] vs 18.7% [95% CI, 15.3%-22.0%]), having never received oral sex compared with having received oral sex (prevalence, 28.6% [95% CI, 23.3%-33.9%] vs 18.0% [95% CI, 14.6%-21.3%]), and having fewer than 4 sexual partners over a lifetime compared with having 4 or more sexual partners (prevalence, 28.1% [95% CI, 22.8%-33.5%] vs 18.4% [95% CI, 14.9%-21.8%]) (Table 4). In bivariate analysis, residing in a metropolitan location was associated with HPV types 16 and 18 compared with residing in a nonmetropolitan location (prevalence, 5.7% [95% CI, 3.1%-8.1%] vs 1.9% [95% CI, 0.8%-3.1%]) (Table 5). In multivariable analyses, the odds of oral HPV-13 or HPV-32 infection was over 2 times higher among those residing in a nonmetropolitan location compared with participants with metropolitan residence (OR, 2.06 [95% CI, 1.10-3.88]) and for participants who had not had a tonsillectomy compared with those who had received the procedure (OR, 2.74; 95% CI, 1.05-7.16) (Table 4). In multivariable analysis, the risk of HPV-16 or HPV-18 infection persisted among women with trade, TAFE, or university education (4.5%; 95% CI, 2.1%-6.9%) (Table 5).

**Table 3.** Bivariate and Multivariable Associations With Prevalence of All Oral HPV Types, Stratified by Sex

Characteristic	All (N = 910)		Men (n = 315)		Women (n = 595)	
	Prevalence, % (95% CI)	OR (95% CI)	Prevalence, % (95% CI)	OR (95% CI)	Prevalence, % (95% CI)	OR (95% CI)
Overall HPV infection	35.3 (32.2-38.4)	NA	33.7 (28.4-38.9)	NA	36.1 (32.3-40.0)	NA
Age group, y						
<37	34.1 (29.7-38.5)	1 [Reference]	33.6 (26.0-41.0)	1 [Reference]	34.4 (28.9-39.9)	1 [Reference]
≥37	36.4 (32.0-40.7)	0.97 (0.58-1.64)	33.7 (26.4-41.0)	0.77 (0.24-2.48)	37.8 (32.3-43.2)	1.09 (0.58-2.05)
Geographic location						
Nonmetropolitan	37.1 (33.1-41.0)	0.81 (0.50-1.31)	36.1 (29.3-43.0)	0.74 (0.25-2.21)	37.5 (32.7-42.4)	0.79 (0.44-1.43)
Metropolitan	32.4 (27.4-37.5)	1 [Reference]	30.1 (21.9-38.2)	1 [Reference]	33.8 (27.4-40.2)	1 [Reference]
Level of education						
Trade/TAFE/university	33.4 (28.0-38.9)	1 [Reference]	28.3 (19.0-37.5)	1 [Reference]	35.9 (29.1-42.7)	1 [Reference]
≤High school	36.3 (32.5-40.2)	0.86 (0.52-1.41)	35.7 (29.4-42.1)	1.15 (0.37-3.59)	36.7 (31.9-41.5)	0.83 (0.44-1.57)
Income						
Job	28.7 (22.7-34.7)	1 [Reference]	28.1 (19.1-37.2)	1 [Reference]	29.2 (21.0-37.3)	1 [Reference]
Centrelink or other	37.5 (34.8-41.1)^a^	1.22 (0.60-2.46)	36.5 (30.1-43.0)	1.23 (0.27-5.65)	37.9 (33.5-42.3)	0.87 (0.34-2.21)
Health care card ownership						
No	32.1 (25.3-38.8)	1 [Reference]	33.3 (22.1-44.5)	1 [Reference]	31.3 (22.8-39.8)	1 [Reference]
Yes	37.0 (33.4-40.6)	1.09 (0.53-2.25)	35.2 (29.0-41.5)	1.19 (0.25-5.71)	37.9 (33.4-42.3)	1.25 (0.48-3.27)
People in house previous night, No.						
≤4	35.4 (31.3-39.5)	1 [Reference]	33.7 (26.8-40.6)	1 [Reference]	36.4 (31.2-41.5)	1 [Reference]
>4	33.4 (28.1-38.7)	0.76 (0.46-1.26)	32.0 (22.6-41.3)	0.44 (0.13-1.53)	34.1 (27.7-40.6)	0.91 (0.49-1.68)
Own car						
No	39.4 (34.6-44.1)	1.28 (0.74-2.23)	36.9 (28.9-44.9)	0.84 (0.21-3.31)	40.7 (34.7-46.6)	1.51 (0.76-2.99)
Yes	32.1 (28.0-36.2)	1 [Reference]	31.0 (24.0-38.0)	1 [Reference]	32.7 (27.6-37.8)	1 [Reference]
Tobacco smoking status						
Never	36.0 (30.0-41.9)	1 [Reference]	33.9 (22.9-44.6)	1 [Reference]	36.9 (29.8-44.0)	1 [Reference]
Current	34.7 (30.6-38.9)	0.64 (0.34-1.22)	33.2 (26.6-39.7)	0.17 (0.04-0.81)	35.8 (30.3-41.2)	1.02 (0.46-2.24)
Former	36.4 (27.3-45.6)	0.99 (0.47-2.10)	37.9 (20.2-55.7)	1.04 (0.20-5.33)	35.9 (25.2-46.6)	1.11 (0.43-2.88)
Alcohol consumption						
Never	36.5 (31.2-41.9)	1 [Reference]	37.5 (26.8-48.2)	1 [Reference]	36.2 (30.0-42.4)	1 [Reference]
Daily	37.5 (20.7-54.3)	0.16 (0.02-1.30)	18.8 (0.0-38.0)	0.18 (0.01-2.76)	56.3 (31.9-80.6)	NS
Weekly	34.3 (27.9-40.7)	0.95 (0.49-1.81)	31.8 (23.1-40.6)	1.18 (0.24-5.74)	36.9 (27.5-46.2)	1.03 (0.46-2.31)
Monthly	34.1 (29.0-39.3)	1.17 (0.66-2.06)	34.0 (24.7-43.3)	0.73 (0.17-3.13)	34.2 (28.0-40.4)	1.54 (0.78 (3.07)
Use of nonprescription tobacco substitutes, eg, vaporizer or e-cigarette						
Never	34.4 (30.6-38.2)	1 [Reference]	32.1 (25.4-38.8)	1 [Reference]	35.5 (30.8-40.1)	1 [Reference]
Current	43.0 (33.3-52.7)	1.35 (0.63-2.92)	37.5 (22.4-52.6)	1.91 (0.31-11.8)	46.7 (34.0-59.3)	0.94 (0.35-2.52)
Former	32.9 (25.7-40.1)	0.90 (0.51-1.58)	35.3 (23.9-46.7)	0.95 (0.27-3.29)	31.3 (21.9-40.6)	0.78 (0.37-1.62)
Use of recreational drugs						
Never	33.7 (29.1-38.4)	1 [Reference]	31.3 (21.9-40.6)	1 [Reference]	34.5 (29.2-39.8)	1 [Reference]
Current	41.8 (34.8-48.8)	1.13 (0.56-2.26)	40.2 (29.9-50.6)	2.16 (0.40-11.6)	43.1 (33.5-52.8)	1.17 (0.50-2.76)
Former	33.1 (27.8-38.4)	1.02 (0.56-1.84)	31 0 (23.0-39.0)	1.35 (0.33-5.59)	34.7 (27.6-41.8)	1.05 (0.50-2.21)
Ever diagnosed with HPV						
No	34.2 (30.7-37.6)	1 [Reference]	31.1 (25.4-36.8)	1 [Reference]	35.8 (31.5-40.1)	1 [Reference]
Yes	22.2 (3.0-41.5)	0.72 (0.21-2.52)	100	NS	17.6 (0.0-35.8)	0.46 (0.11-1.92)
Don’t know	41.6 (33.8-49.4)	0.63 (0.32-1.22)	42.9 (29.8-55.9)	0.33 (0.06-1.78)	40.8 (31.1-50.6)	0.58 (0.26-1.30)
Ever received HPV vaccination						
Yes	28.0 (17.8-38.2)	1 [Reference]	42.9 (6.0-79.7)	1 [Reference]	26.5 (16.0-37.0)	1 [Reference]
No	36.6 (32.5-40.8)	1.55 (0.65-3.70)	33.5 (26.9-40.1)	0.78 (0.01-52.4)	38.5 (33.2-43.8)	1.68 (0.64-4.39)
Don’t know	34.4 (29.1-39.7)	1.87 (0.78-4.47)	32.4 (23.5-41.3)	1.21 (0.02-86.7)	35.5 (28.8-42.2)	2.23 (0.86-5.77)
Tonsils removed						
Yes	33.3 (24.7-42.0)	1 [Reference]	36.7 (19.3-54.0)	1 [Reference]	32.1 (22.1-42.2)	1 [Reference]
No	35.0 (31.5-38.5)	0.79 (0.43-1.46)	33.1 (27.1-39.0)	0.48 (0.12-1.88)	36.0 (31.7-40.3)	0.85 (0.39-1.86)
Don’t know	41.3 (27.0-55.6)	0.86 (0.20-3.60)	35.7 (17.9-53.6)	0.64 (0.06-7.56)	50.0 (26.8-73.2)	0.78 (0.09-7.24)
In life, how many passionately kissed						
<4	37.5 (31.8-43.1)	1 [Reference]	38.8 (28.4-49.2)	1 [Reference]	36.9 (30.1-43.6)	1 [Reference]
≥4	33.1 (29.1-37.1)	1.07 (0.50-2.27)	30.4 (23.9-36.9)	1.54 (0.25-9.65)	34.7 (29.6-39.8)	0.87 (0.34-2.20)
Ever given oral sex						
Yes	33.3 (29.3-37.4)	NS	29.4 (22.6-36.1)	NS	35.3 (30.3-40.4)	NS
No	35.8 (30.2-41.3)	NS	37.5 (28.1-46.9)	NS	34.8 (27.9-41.7)	NS
If yes, age when first gave oral sex, y						
≥16	31.5 (26.9-36.2)	1 [Reference]	26.4 (18.6-34.2)	1 [Reference]	34.0 (28.2-39.7)	1 [Reference]
<16	38.6 (30.1-47.1)	1.36 (0.50-2.27)	36.0 (22.6-49.4)	0.72 (0.18-2.81)	40.3 (29.3-51.3)	2.35 (0.77-7.22)
People given oral sex to in lifetime, No.						
≤3	31.5 (26.1-36.9)	1 [Reference]	27.8 (17.9-37.8)	1 [Reference]	32.9 (26.5-39.2)	1 [Reference]
>3	35.7 (29.4-41.9)	1.56 (0.76-3.17)	30.5 (21.2-39.9)	1.38 (0.36-5.28)	39.3 (31.0-47.5)	1.09 (0.35-3.34)
Ever received oral sex						
Yes	32.8 (28.8-36.9)	NS	27.5 (21.1-33.9)	NS	35.9 (30.7-41.1)	NS
No	37.1 (31.5-42.7)	NS	42.7 (32.3-53.0)	NS	34.5 (27.8-41.2)	NS
If yes, age when first received oral sex, y						
≥16	31.7 (26.9-36.5)	1 [Reference]	22.0 (14.2-29.9)	1 [Reference]	35.8 (29.9-41.7)	1 [Reference]
<16	34.5 (26.7-42.2)	0.89 (0.42-1.90)	35.4 (24.8-46.1)	1.22 (0.28-5.26)	33.3 (21.9-44.8)	0.52 (0.17-1.59)
People received oral sex from in lifetime, No.						
≤3	30.4 (24.7-36.0)	1 [Reference]	20.3 (10.0-30.7)	1 [Reference]	33.3 (26.7-39.9)	1 [Reference]
>3	34.8 (28.9-40.6)	1.29 (0.62-2.69)	31.0 (23.0-39.1)	1.22 (0.21-7.25)	38.5 (30.1-47.1)	2.37 (0.75-7.49)
Sexual intercourse with another person						
Yes	34.6 (31.2-38.0)	NS	32.2 (26.5-37.9)	NS	35.9 (31.6-40.1)	NS
No	31.0 (16.9-45.0)	NS	30.7 (5.5-56.0)	NS	31.0 (14.1-47.9)	NS
If yes, age when first had sex, y						
≥16	34.1 (29.8-38.5)	NS	27.7 (20.2-35.3)	NS	36.9 (31.6-42.3)	NS
<16	36.2 (30.8-38.5)	1.14 (0.61-2.12)	37.4 (28.7-46.0)	3.68 (0.90-15.0)	35.4 (28.3-42.4)	0.83 (0.38-1.78)
Sexual partners						
≥4	33.2 (29.0-37.4)	0.66 (0.31-1.38)	31.1 (24.5-37.7)	0.55 (0.06-4.88)	34.6 (29.1-40.1)	0.69 (0.29-1.68)
<4	37.0 (31.3-42.8)	NS	35.9 (24.1-47.8)	NS	37.4 (30.7-44.0)	NS
In lifetime, sexual encounters have been mainly						
Heterosexual	35.0 (31.6-38.4)	1 [Reference]	32.2 (26.5-37.9)	1 [Reference]	36.5 (32.2-40.7)	1 [Reference]
Homosexual	50.0 (9.9-90.1)	0.62 (0.03-12.2)	25.0 (0.0-67.7)	0.59 (0.02-21.9)	100	NA
Bisexual	26.7 (13.7-39.6)	0.44 (0.18-1.06)	37.5 (3.7-71.3)	0.46 (0.03-7.77)	24.3 (10.5-38.2)	0.37 (0.13-1.00)
Current relationship status						
Stable long-term	35.4 (30.7-40.0)	1 [Reference]	32.1 (24.2-40.0)	1 [Reference]	37.0 (31.2-42.7)	1 [Reference]
Short-term	31.9 (29.1-39.1)	1.16 (0.44-0.18)	36.4 (7.7-65.0)	4.04 (0.47-34.7)	30.6 (15.5-45.7)	0.84 (0.26-2.74)
Single	34.1 (29.1-39.1)	0.96 (0.58-1.59)	32.8 (24.6-41.0)	1.26 (0.41-3.92)	34.8 (28.6-41.1)	1.00 (0.53-1.88)

Abbreviations: HPV, human papillomavirus; NA, not applicable; NS, not significant (odds ratio for this variable is either less than 0.000 or more than 999.99); OR, odds ratio.

^a^Results were significant using a χ^2^ test at the level of *P* < .05.

**Table 4.** Bivariate and Multivariable Associations With Prevalence of Oral HPV Types 13 and 32, Stratified by Sex

Characteristic	All (N = 910)		Men (n = 315)		Women (n = 595)	
	Prevalence, % (95% CI)	OR (95% CI)	Prevalence, % (95% CI)	OR (95% CI)	Prevalence, % (95% CI)	OR (95% CI)
Overall HPV-13 or HPV-32 infection	22.7 (20.0-25.5)	NA	19.0 (14.7-23.4)	NA	24.7 (21.2-28.2)	NA
Age, y						
<37	22.7 (18.8-26.7)	1 [Reference]	21.7 (15.1-28.3)	1 [Reference]	23.3 (18.4-28.2)	1 [Reference]
≥37	22.8 (19.0-26.6)	0.71 (0.36-1.40)	16.6 (10.8-22.3)	0.71 (0.21-2.34)	26.1 (21.1-31.0)	0.89 (0.42-1.90)
Geographic location						
Metropolitan	13.1 (9.5-16.7)	1 [Reference]	9.8 (4.5-15.0)	1 [Reference]	15.0 (10.2-19.8)	1 [Reference]
Nonmetropolitan	28.5 (24.8-32.2)^a^	2.06 (1.10-3.88)^a^	25.1 (18.9-31.3)^a^	2.80 (0.80-9.79)	30.2 (25.6-34.8)^a^	1.82 (0.88-3.76)
Level of education						
Trade/TAFE/university	18.8 (14.3-23.30	1 [Reference]	12.0 (5.3-18.6)	1 [Reference]	22.1 (16.2-27.9)	1 [Reference]
≤High school	24.7 (21.3-28.1)	1.08 (0.57-2.07)	22.2 (16.7-27.7)	1.31 (0.36-4.78)	26.2 (21.8-30.5)	1.05 (0.50-2.24)
Income						
Job	19.0 (13.7-24.2)	1 [Reference]	16.7 (9.2-24.2)	1 [Reference]	20.8 (13.5-28.1)	1 [Reference]
Centrelink or other	23.9 (20.7-27.1)	1.21 (0.49-2.99)	20.4 (15.0-25.8)	0.62 (0.14-2.79)	25.5 (21.5-29.4)	0.98 (0.33-2.88)
Health care card ownership						
No	21.7 (15.8-27.7)	1 [Reference]	20.3 (10.7-29.8)	1 [Reference]	22.6 (14.9-30.3)	1 [Reference]
Yes	20.9 (20.1-26.4)	0.75 (0.30-1.88)	18.9 (13.8-24.1)	0.70 (0.14-3.58)	25.3 (21.4-29.4)	0.89 (0.2-2.75)
People in house previous night, No.						
<4	21.5 (18.0-25.0)	1 [Reference]	17.4 (11.9-22.9)	1 [Reference]	23.8 (19.2-28.3)	1 [Reference]
≥4	24.9 (20.1-29.8)	0.94 (0.50-1.76)	21.6 (13.4-29.9)	0.82 (0.24-2.88)	26.4 (20.4-32.5)	1.01 (0.50-2.07)
Own car						
Yes	18.5 (15.1-21.9)	1 [Reference]	14.0 (8.8-19.3)	1 [Reference]	20.8 (16.4-25.2)	1 [Reference]
No	28.0 (23.6-32.4)^a^	1.02 (0.51-2.03)	24 8 (17.7-32.0)	1.20 (0.33-4.38)	29.7 (24.1-35.2)	1.19 (0.55-2.61)
Tobacco smoking						
Never	24.1 (18.8-29.4)	1 [Reference]	18.9 (9.9-27.9)	1 [Reference]	26.3 (19.8-32.7)	1 [Reference]
Current	23.4 (19.6-27.1)	1.26 (0.57-2.82)	20.1 (14.5-25.7)	0.70 (0.15-3.21)	25.5 (20.6-30.4)	1.57 (0.61-4.06)
Former	15.0 (8.2-21.7)	0.82 (0.28-2.36)	10.3 (0.0-21.5)	0.94 (0.14-6.44)	16.7 (8.4-25.0)	0.72 (0.20-2.60)
Alcohol consumption						
Never	22.8 (18.1-27.4)	1 [Reference]	22.5 (13.3-31.7)	1 [Reference]	22.8 (17.4-28.3)	1 [Reference]
Daily	28.1 (12.5-43.7)	NS	6.3 (0.0-18.2)	NS	50.0 (25.4-74.6)	NS
Weekly	21.1 (15.6-26.6)	1.17 (0.51-2.69)	18.2 (10.9-25.4)	NS	24.3 (16.0-32.6)	1.04 (0.39-2.77)
Monthly	22.6 (18.0-27.1)	1.57 (0.77-3.21)	18.0 (10.4-25.6)	NS	24.6 (19.0-30.2)	1.89 (0.84-4.26)
Use of nonprescription tobacco substitutes, eg, vaporizer or electronic cigarette						
Never	23.5 (20.1-26.9)	1 [Reference]	19.5 (13.8-25.1)	NS	25.4 (21.1-29.6)	1 [Reference]
Current	27.0 (18.3-35.7)	1.03 (0.40-2.63)	17.5 (5.6-29.3)	NS	33.3 (21.4-45.3)	0.79 (0.25-2.50)
Former	17.1 (11.3-22.8)	0.46 (0.21-1.02)	19.1 (9.7-28.5)	NS	15.6 (8.3-22.9)	0.41 (0.16-1.090
Use of recreational drugs						
Never	24.9 (20.7-29.1)	1 [Reference]	20.8 (12.7-29.0)	1 [Reference]	26.1 (21.2-31.0)	1 [Reference]
Current	25.9 (19.7-32.2)	0.71 (0.30-1.66)	25.3 (16.1-34.5)	1.48 (0.29-7.58)	26.5 (17.9-35.1)	0.72 (0.26-1.99)
Former	17.2 (12.9-21.5)	0.55 (0.26-1.17)	13.2 (7.3 -19.0)	0.44 (0.09-2.26)	20.2 (14.2-26.2)	0.65 (0.27-1.57)
Ever diagnosed with HPV						
No	21.8 (18.8-24.8)	1 [Reference]	16.5 (11.9-21.1)	NS	24.6 (20.7-28.5)	1 [Reference]
Yes	11.1 (0.0-25.7)	0.56 (0.1-3.01)	0	NS	11.8 (0.0-27.1)	0.37 (0.06-2.16)
Don’t know	28.6 (21.4-35.7)	0.71 (0.31-1.62)	30.4 (18.2-42.5)	NS	27.6 (18.7-36.4)	0.57 (0.21-1.53)
Ever received HPV vaccination						
Yes	17.3 (8.7-25.9)	1 [Reference]	28.6 (0.0-62.2)	NS	16.2 (7.4-25.0)	NS
No	24.3 (20.6-28.0)	1.53 (0.52-4.51)	18.8 (13.3-24.3)	NS	27.6 (22.7-32.5)	1.39 (0.45-4.29)
Don’t know	21.4 (16.8-26.0)	1.69 (0.58-4.94)	18.5 (11.2-25.9)	NS	23.0 (17.2-28.8)	1.77 (0.59-5.35)
Tonsils removed						
Yes	14.0 (7.6-20.4)^a^	1 [Reference]	6.7 (0.0-15.6)	NS	16.7 (8.7-24.7)	1 [Reference]
No	24.1 (20.9-27.2)	2.74 (1.05-7.16)^a^	21.2 (16.1-26.4)	NS	25.5 (21.6-29.4)	2.09 (0.72-6.04)
Don’t know	23.9 (11.6-36.3)	1.76 (0.25-12.5)	17.9 (3.6-32.1)	NS	33.3 (11.5-55.2)	0.69 (0.04-12.9)
In life, how many passionately kissed						
≥4	19.7 (16.3-23.0)	0.81 (0.33-1.97)	14.9 (9.9-20.0)	0.32 (0.08-1.29)	22.4 (17.9-26.8)	0.91 (0.32-2.61)
<4	26.9 (21.7-32.0)	NA	28.2 (18.6-37.9)	NA	26.3 (20.1-32.4)	NA
Ever given oral sex						
Yes	18.7 (15.3-22.0)^a^	NS	11.3 (6.6-16.0)^a^	NS	22.4 (18.0-26.8)	NS
No	27.4 (22.3-32.6)	NS	30.8 (21.8-39.7)	NS	25.5 (19.2-31.9)	NS
If yes, age when first gave oral sex, y						
≥16	17.2 (13.4-20.9)	1 [Reference]	9.6 (4.3-14.8)	NS	20.8 (15.8-25.7)	1 [Reference]
<16	23.6 (16.2-31.0)	1.64 (0.61-4.40)	16.0 (5.7-26.3)	NS	28.6 (18.4-38.7)	2.50 (0.65-9.64)
People given oral sex to in lifetime, No.						
>3	17.0 (12.1-21.8)	2.07 (0.83-5.16)	10.5 (4.3-16.8)	1.58 (0.43-5.73)	21.5 (14.5-28.4)	1.53 (0.39-6.00)
≤3	20.1 (15.4-24.7)	NA	12.7 (5.3-20.1)	NA	22.9 (17.1-28.6)	NA
Ever received oral sex						
Yes	18.0 (14.6-21.3)^a^	NS	10.1 (5.7-14.4)^a^	NS	22.5 (18.0-27.0)	NS
No	28.6 (23.3-33.9)	NS	36.0 (25.9-46.0)	NS	25.3 (19.1-31.4)	NS
If yes, age when first received oral sex, y						
≥16	18.3 (14.3-22.3)	1 [Reference]	9.2 (3.7-14.6)	NS	22.2 (17.1-27.3)	1 [Reference]
<16	15.2 (9.3-21.0)	0.52 (0.19-1.44)	11.4 (4.3-18.5)	NS	19.7 (10.1-29.3)	0.45 (0.12-1.73)
People received oral sex from in lifetime, No.						
≤3	19.8 (15.0-24.7)	1 [Reference]	8.5 (1.3-15.6)	NS	23.2 (17.3-29.1)	1 [Reference]
>3	15.2 (10.8-19.7)	0.83 (0.33-2.13)	10.9 (5.4-16.3)	NS	19.7 (12.7-26.6)	1.29 (0.32-5.21)
Sexual intercourse with another person						
Yes	22.0 (19.0-24.9)	NS	18.0 (13.3-22.7)	NS	24.0 (20.3-27.8)	NS
No	19.0 (7.1-30.9)	NS	23.1 (0.0-46.1)	NS	17.2 (3.4-31.0)	NS
If yes, age when first had sex, y						
≥16	22.2 (18.3-26.0)	1 [Reference]	16.8 (10.5-23.1)	1 [Reference]	24.5 (19.7-29.3)	1 [Reference]
<16	22.3 (17.5-27.0)	1.17 (0.54-2.52)	19.5 (12.5-26.6)	NS	24.2 (17.8-30.5)	0.90 (0.37-2.19)
Sexual partners						
<4	28.1 (22.8-33.5)	1 [Reference]	29.7 (18.4-41.0)	NS	27.7 (21.5-33.8)	1 [Reference]
≥4	18.4 (14.9-21.8)^a^	0.77 (0.31-1.94)	14.0 (9.1-18.9)	NS	21.3 (16.6-26.1)	0.79 (0.28-2.19)
In lifetime, sexual encounters have been mainly						
Heterosexual	22.3 (19.4-25.3)	1 [Reference]	18.4 (13.7-23.1)	NS	24.4 (20.6-28.3)	1 [Reference]
Homosexual	33.3 (0.0-71.1)	NS	0	NS	100	NS
Bisexual	11.1 (1.9-20.3)	0.53 (0.116-1.75)	25.0 (0.0-55.2)	NS	8.1 (0.0-16.9)	0.30 (0.07-1.22)
Current relationship status						
Stable long-term	22.3 (18.3-26.3)	1 [Reference]	19.7 (13.0-26.4)	1 [Reference]	23.6 (18.5-28.6)	1 [Reference]
Short-term	21.3 (9.6-33.0)	1.06 (0.33-3.46)	18.2 (0.0-41.1)	0.67 (0.04-11.4)	22.2 (8.6-35.8)	1.00 (0.26-3.89)
Single	21.3 (17.0-25.6)	0.90 (0.48-1.70)	17.2 (10.6-23.8)	0.80 (0.24-2.66)	23.7 (18.1-29.2)	1.02 (0.48-2.17)

Abbreviations: HPV, human papillomavirus; NA, not applicable; NS, not significant (odds ratio for this variable either less than 0.000 or greater than 999.99); OR, odds ratio.

^a^ Results were significant using a χ^2^ test at the level of *P* < .05.

**Table 5.** Bivariate and Multivariable Associations With Prevalence of Oral HPV Types 16 and 18, Stratified by Sex

Characteristic	All (N = 910)		Men (n = 315)		Women (n = 595)	
	Prevalence, % (95% CI)	OR (95% CI)	Prevalence, % (95% CI)	OR (95% CI)	Prevalence, % (95% CI)	OR (95% CI)
Overall HPV-16 or HPV-18 infection	3.3 (2.1-4.5)	NA	2.9 (1.0-4.7)	NA	3.5 (2.0-5.0)	NA
Age, y						
<37	3.4 (1.7-5.1)	1 [Reference]	2.6 (0.1-5.1)	1 [Reference]	2.9 (1.6-6.0)	1 [Reference]
≥37	3.2 (1.6-4.8)	0.72 (0.13-4.05)	3.1 (0.4-5.7)	2.61 (0.23-29.8)	3.3 (1.3-5.2)	0.48 (0.08-2.93)
Geographic location						
Metropolitan	5.7 (3.1-8.1)	1 [Reference]	2.4 (0.0-5.2)	1 [Reference]	7.5 (4.0-11.1)	1 [Reference]
Nonmetropolitan	1.9 (0.8-3.1)^a^	0.18 (0.02-1.34)	3.1 (0.7-5.6)	0.29 (0.03-2.89)	1.3 (0.2-2.5)^a^	0.53 (0.10-2.77)
Level of education						
Trade/TAFE/university	4.5 (2.1-6.9)	1 [Reference]	3.3 (0.0-6.9)	1 [Reference]	5.1 (2.0-8.2)	1 [Reference]
≤High school	2.8 (1.5-4.1)	0.40 (0.08-2.02)	2.7 (0.6-4.9)	0.72 (0.05-10.1)	2.8 (1.2-4.5)	0.16 (0.03-0.97)^a^
Income						
Job	2.3 (0.3-4.3)	1 [Reference]	3.1 (0.0-6.6)	1 [Reference]	1.7 (0.0-4.0)	1 [Reference]
Centrelink or other	3.7 (2.2-5.1)	1.72 (0.21-14.0)	2.8 (0.6-5.0)	0.41 (0.03-5.70)	4.1 (2.3-5.9)	6.19 (0.35-108.5)
Health care card ownership						
No	1.6 (0.0-3.5)	1 [Reference]	2.9 (0.0-6.9)	1 [Reference]	0.9 (0.0-2.6)	1 [Reference]
Yes	3.8 (2.4-5.2)	5.80 (0.41-82.4)	3.1 (0.8-5.3)	6.06 (0.19-198.8)	4.2 (2.3-6.0)	2.03 (0.10-40.7)
People in house previous night, No.						
<4	3.8 (2.2-5.5)	1 [Reference]	3.3 (0.7-5.8)	1 [Reference]	4.1 (2.0-6.2)	1 [Reference]
≥4	1.6 (0.2-3.1)	0.26 (0.03-2.50)	3.1 (0.0-6.6)	0.46 (0.03-6.96)	1.0 (0.0-2.3)	0.19 (0.02-2.00)
Car ownership						
Yes	3.8 (2.1-5.5)	1 [Reference]	4.7 (1.5-7.9)	1 [Reference]	3.4 (1.4-5.3)	1 [Reference]
No	2.7 (1.1-4.3)	0.46 (0.07-3.23)	0.7 (0.0-2.1)	NS	3.8 (1.5-6.1)	0.64 (0.09-4.58)
Tobacco smoking						
Never	4.3 (1.8-6.9)	1 [Reference]	5.4 (0.2-10.6)	1 [Reference]	3.9 (1.1-6.8)	1 [Reference]
Current	2.0 (0.8-3.2)	0.67 (0.08-5.95)	1.5 (0.0-3.2)	1.61 (0.12-21.7)	2.3 (0.6-4.0)	0.28 (0.04-2.04)
Former	6.5 (1.8-11.2)	2.05 (0.23-18.4)	3.4 (0.0-10.1)	2.27 (0.09-55.6)	7.7 (1.8-13.6)	0.80 (0.09-6.92)
Alcohol consumption						
Daily	0	NS	0	NS	0	NS
Weekly	2.8 (0.6-5.0)	NS	2.7 (0.0-5.8)	NS	2.9 (0.0-6.2)	NS
Monthly	4.6 (2.3-6.8)	NS	5.0 (0.7-9.3)	NS	4.4 (1.7-7.1)	NS
Never	2.9 (1.0-4.7)	NS	1.3 (0.0-3.7)	NS	3.4 (1.1-5.8)	NS
Use of nonprescription tobacco substitutes, eg, vaporizer or electronic cigarette						
Current	2.0 (0.0-4.7)	NS	0	NS	3.3 (0.0-7.9)	NS
Former	4.3 (1.2-7.4)	NS	5.9 (0.3-11.5)	NS	3.1 (0.0-6.6)	NS
Never	3.4 (1.9-4.8)	NS	2.6 (0.3-4.9)	NS	3.7 (1.9-5.5)	NS
Use of recreational drugs						
Current	2.1 (0.1-4.2)	NA	1.1 (0.0-3.4)	NA	2.9 (0.0-6.2)	NA
Former	4.6 (2.3-7.0)	NA	3.9 (0.5-7.2)	NA	5.2 (1.9-8.5)	NA
Never	3.0 (1.3-4.6)	NA	3.1 (0.0-6.6)	NA	2.9 (1.0-4.8)	NA
Ever diagnosed with HPV						
Yes	5.6 (0.0-16.2)	1 [Reference]	100	NS	0	NS
No	3.3 (2.0-4.6)	6.83 (0.46-101.3)	2.4 (0.5-4.2)	NS	3.8 (2.1-5.5)	NS
Don’t know	3.2 (0.4-6.1)	0.59 (0.04-9.07)	3.6 (0.0-8.5)	NS	3.1 (0.0-6.5)	NS
Ever received HPV vaccination						
Don’t know	3.6 (1.5-5.6)	1 [Reference]	2.8 (0.0-5.9)	NA	4.0 (1.3-6.7)	1 [Reference]
Yes	5.3 (0.2-10.4)	1.07 (0.07-17.3)	0	NS	5.9 (0.3-11.5)	0.22 (0.02-2.66)
No	2.9 (1.4-4.3)	0.89 (0.05-15.2)	3.0 (0.6-5.5)	NS	2.8 (1.0-4.6)	0.66 (0.08-5.73)
Tonsils removed						
Yes	3.5 (0.1-6.9)	1 [Reference]	0	NS	4.8 (0.2-9.3)	NS
No	2.9 (1.7-4.1)	0.32 (0.06-1.81)	2.4 (0.5-4.4)	NS	3.1 (1.6-4.7)	NS
Don’t know	8.7 (0.5-16.9)	2.51 (0.08-82.5)	10.7 (0.0-22.2)	NS	5.6 (0.0-16.2)	NS
In life, how many passionately kissed						
<4	3.5 (1.4-5.7)	1 [Reference]	2.4 (0.0-5.6)	1 [Reference]	4.0 (1.3-6.8)	1 [Reference]
≥4	3.0 (1.5-4.4)	0.55 (0.02-13.1)	2.6 (0.3-4.8)	0.90 (0.07-12.4)	3.2 (1.3-5.1)	0.54 (0.04-6.48)
Ever given oral sex						
Yes	3.2 (1.1-5.1)	NS	2.8 (0.4-5.3)	NS	3.4 (1.5-5.3)	NS
No	3.1 (1.1-5.1)	NS	1.9 (0.0-4.6)	NS	3.8 (1.0-6.6)	NS
If yes, age when first gave oral sex, y						
≥16	3.6 (1.7-5.4)	1 [Reference]	2.4 (0.0-5.1)	1 [Reference]	4.2 (1.7-6.6)	1 [Reference]
<16	2.4 (0.0-5.0)	0.36 (0.05-2.67)	4.0 (0.0-9.5)	2.19 (0.20-24.3)	1.3 (0.0-3.8)	0.72 (0.04-12.9)
People given oral sex to in lifetime, No.						
≤3	3.1 (1.1-5.1)	1 [Reference]	2.5 (0.0-6.0)	1 [Reference]	3.3 (0.9-5.8)	1 [Reference]
>3	3.5 (1.1-5.9)	3.29 (0.42-25.8)	3.2 (0.0-6.7)	1.94 (0.12-31.4)	3.7 (0.5-6.9)	1.64 (0.26-10.31)
Ever received oral sex						
Yes	2.9 (1.4-4.3)	NS	2.6 (0.3-4.9)	NS	3.0 (1.2-4.9)	NS
No	3.9 (1.6-6.1)	NS	2.2 (0.0-5.3)	NS	4.6 (1.7-7.6)	NS
If yes, age when first received oral sex, y						
≥16	2.5 (0.9-4.1)	1 [Reference]	0.9 (0.0-2.7)	NS	3.1 (1.0-5.2)	NS
<16	4.1 (0.9-7.4)	6.59 (0.90-48.3)	5.1 (0.2-9.9)	NS	3.0 (0.0-7.2)	NS
People received oral sex from in lifetime, No.						
>3	4.3 (1.8-6.8)	NS	3.9 (0.5-7.2)	NS	4.7 (1.0-8.4)	NS
≤3	1.6 (0.0-3.1)	NS	0	NS	2.0 (0.1-4.0)	NS
Sexual intercourse with another person						
Yes	3.2 (1.9-4.4)	NS	2.7 (0.7-4.7)	NS	3.4 (1.8-5.0)	NS
No	4.8 (0.0-11.2)	NS	0	NS	6.9 (0.0-16.1)	NA
If yes, age when first had sex, y						
≥16	3.8 (2.0-5.5)	NS	3.6 (0.5-6.9)	1 [Reference]	3.8 (1.7-5.9)	1 [Reference]
<16	2.3 (0.6-4.0)	NS	1.6 (0.0-3.9)	0.30 (0.02-4.29)	2.8 (0.4-5.2)	0.52 (0.05-5.00)
Sexual partners						
<4	3.0 (0.9-5.0)	1 [Reference]	1.6 (0.0-4.6)	1 [Reference]	3.4 (0.9-5.9)	1 [Reference]
≥4	3.3 (1.7-5.0)	1.06 (0.04-30.7)	3.1 (0.6-5.6)	0.23 (0.01-5.96)	3.5 (1.4-5.6)	1.71 (0.14-20.8)
In lifetime, sexual encounters have been mainly						
Heterosexual	3.5 (2.1-4.8)	NS	2.7 (0.7-4.7)	NS	3.9 (2.2-5.6)	1 [Reference]
Homosexual	0	NS	0	NS	0	NS
Bisexual	2.2 (0.0-6.5)	NS	0	NS	2.7 (0.0-7.9)	0.63 (0.04-9.05)
Current relationship status						
Stable long-term	3.4 (1.6-5.1)	1 [Reference]	2.2 (0.0-4.7)	1 [Reference]	4.0 (1.7-6.3)	NS
Short-term	2.1 (0.0-6.3)	1.76 (0.11-28.2)	9.1 (0.0-26.2)	5.59 (0.25-126.6)	0	NS
Single	3.4 91.5-5.3)	0.89 (0.18-4.46)	2.3 (0.0-5.0)	0.18 (0.01-2.72)	4.0 (1.4-6.6)	NS

Abbreviations: HPV, human papillomavirus; NA, not applicable; NS, not significant (odds ratio for this variable is either less than 0.000 or greater than 999.9); OR, odds ratio.

^a^ Results were significant using a χ^2^ test at the level of *P* < .05.

## Discussion

Consistent with our hypothesis, the prevalence of any oral HPV type, HPV types associated with Heck disease (HPV-13 and HPV-32), and HPV types associated with OPSCC (HPV-16 and HPV-18) among Indigenous Australians appeared to be higher than those reported both in other Australian studies and in populations from other countries. The prevalence of oral HPV in the current study was 15.3 times that reported in a study of young non–Indigenous Australians^24^ and 4.7 times that reported by Antonsson et al^23^ in a systematic review involving 9 studies from other countries.

The prevalence of HPV-13 or HPV-32 was 0 in the Australian study. The systematic review reported no prevalence estimates for HPV-13 or HPV-32, but it is unclear if that is because the prevalence was 0 or if these estimates were not analyzed. The prevalence of HPV types associated with OPSCC in the current study was 2.5 times that reported in the Australian study and 2.1 times that reported in the review of studies from other countries.

Our additional hypothesis that risk factors would include male sex, social disadvantage, tobacco use, and early and high levels of sexual activity proved only partially true. Indicators of social disadvantage were associated with Heck disease, but so were low rates of sexual activity. There were no apparent risk factors for HPV types associated with OPSCC aside from residing in a metropolitan location. The prevalence of HPV among those having given oral sex (64.6%) was much lower in the current study than in the second Australian Study of Health and Relationships (77.0%).^31^

The high levels of oral carriage of HPV in our study are concerning, particularly the high prevalence of HPV types associated with OPSCC. It is particularly interesting that the rates were higher among women, across both younger and older demographic characteristics. The findings speak to an urgent need to ensure high HPV vaccination coverage in Indigenous adolescents (although this does not prevent infection with HPV-13 and HPV-32), particularly given the evidence of HPV vaccine efficacy in decreasing the subsequent prevalence of oral HPV infection.^32^ Further research could assess the effectiveness and cost-effectiveness of immunization of those in older age groups (ie, those aged 20 years or older who are no longer eligible for free vaccination). Efforts to extend the benefits provided by vaccination would need to take into account the lower vaccine effectiveness among those already exposed to HPV and the long latent period between a causal HPV infection and invasive disease. Both the effectiveness of vaccination against persistent oral HPV infection at older ages and its cost-effectiveness are yet to be demonstrated. Australian adolescents aged up to 19 years can receive 2 doses of the HPV vaccine free of charge as part of the National HPV Vaccination Program, and HPV vaccination is also available in Australia for women aged 20 to 45 years and men aged 20 to 26 years but is not reimbursed in the public program. Evidence has shown that the level of elective uptake in women not eligible for vaccination through the publicly funded program in Australia is low (ie, 11%).^33^

The rates of oral carriage of HPV types associated with Heck disease in our study were higher than what has been presented in the literature to date. For example, in the Australian study among university students, there were no HPV-13 or HPV-32 types identified.^24^ Although factors that determine susceptibility for Heck disease are unclear, genetic susceptibility—especially concerning the human lymphocytic antigen (HLA)-DR4(DRB1*0404) allele (an allele occurring frequently in Indigenous populations of the Americas but with no documented reports among Indigenous Australians)—is thought to play a major role in vulnerability to HPV-13 and HPV-32.^29^ It is reassuring that HPV types associated with Heck disease are considered low risk (ie, they are not found in cancers), with spontaneous regression of clinical lesions during a mean of 18 months.^34^ It is perhaps unsurprising that associations with HPV-13 and HPV-32 in our study included low sexual activity, given that Heck disease is not associated with high sexual activity.^34^ The hypothesized mode of transition is horizontal (mouth-to-mouth), commencing early in infancy via the mother.^35^

The critical issue with high-risk oral HPV infections regarding OPSCC (or any HPV-related head and neck cancer) is persistent oral HPV infection. In a large cohort study of incidence and clearance of oral HPV among men who did not have HIV or anogenital cancer, Kreimer et al^36^ reported that during the first 12 months of follow-up, 4.4% of men acquired an incident oral HPV infection, with 1.7% of this 4.4% being an oncogenic HPV type and 0.6% of the 4.4% being HPV-16. Acquisition of oral oncogenic HPV was significantly associated with tobacco smoking and being single and was similar across included countries, age groups, and reported sexual behaviors. The median duration of infection was 6.9 months for any oral HPV, 6.3 months for oncogenic HPV, and 7.3 months for HPV-16. Eight of the 18 incident oral HPV-16 infections (44.4%) persisted for 6 or more months. The authors concluded that newly acquired oral oncogenic HPV infections in healthy men were rare and that most cleared within 1 year. The incidence, clearance, or persistence of oral HPV infections among Indigenous Australians remains unknown and is the subject of continuing research.

### Limitations

This study has limitations. It did not include clinical dental examinations, which would have revealed any physical manifestations of both Heck disease and early-stage OPSCC. The study was not representative, with almost two-thirds of participants being women. Oral carriage of HPV is usually higher among men, and because only 35% of participants in our study were men, our findings may underestimate the true prevalence of oral HPV in the Indigenous population. The difficulties in recruiting men to health-related studies is widely documented,^37,38^ including in the Indigenous Australian context.^39^ We found no association between sex and HPV infection in our sample. Conversely, given that we found that higher HPV prevalence was associated with living in nonmetropolitan areas and that people living in these areas were overrepresented in our sample compared with the Indigenous population in South Australia, this may have overestimated prevalence. In direct comparisons with other studies, this study did not age match.

## Conclusions

In this study, the overall prevalence of HPV detected in oral fluid in a large convenience sample of Indigenous Australians was high, with one-third demonstrating carriage on a single occasion. The most prevalent HPV types were those associated with Heck disease (HPV-13 and HPV-32). The next most prevalent were types most strongly associated with OPSCC (HPV-16 and HPV-18). Prevalence of these types appeared to exceed both Australian and international population-level estimates.

## References

[1] Lechner M, Breeze CE, O’Mahony JF, Masterson L Early detection of HPV-associated oropharyngeal cancer. Lancet. 2019;393(10186):2123. doi:10.1016/S0140-6736(19)30227-2 31226048

[2] D’Souza G, Kreimer AR, Viscidi R, Case-control study of human papillomavirus and oropharyngeal cancer. N Engl J Med. 2007;356(19):1944-1956. doi:10.1056/NEJMoa065497 17494927

[3] Hong A, Lee CS, Jones D, Rising prevalence of human papillomavirus-related oropharyngeal cancer in Australia over the last 2 decades. Head Neck. 2016;38(5):743-750. doi:10.1002/hed.23942 25521312

[4] Plummer M, de Martel C, Vignat J, Ferlay J, Bray F, Franceschi S Global burden of cancers attributable to infections in 2012: a synthetic analysis. Lancet Glob Health. 2016;4(9):e609-e616. doi:10.1016/S2214-109X(16)30143-7 27470177

[5] Castellsagué X, Alemany L, Quer M, ; ICO International HPV in Head and Neck Cancer Study Group HPV involvement in head and neck cancers: comprehensive assessment of biomarkers in 3680 patients. J Natl Cancer Inst. 2016;108(6):djv403. doi:10.1093/jnci/djv403 26823521

[6] Yakin M, Seo B, Hussaini H, Rich A, Hunter K Human papillomavirus and oral and oropharyngeal carcinoma: the essentials. Aust Dent J. 2019;64(1):11-18. doi:10.1111/adj.12652 30238467

[7] National Cancer Institute Annual report to the nation on the status of cancer. Accessed September 30, 2019. https://seer.cancer.gov/report_to_nation/

[8] Office for National Statistics Cancer registration statistics, England: first release, 2016 Accessed September 30, 2019. https://www.ons.gov.uk/peoplepopulationandcommunity/healthandsocialcare/conditionsanddiseases/bulletins/cancerregistrationstatisticsengland/2016

[9] Henke RP, Guèrin-Reverchon I, Milde-Langosch K, Koppang HS, Löning T In situ detection of human papillomavirus types 13 and 32 in focal epithelial hyperplasia of the oral mucosa. J Oral Pathol Med. 1989;18(7):419-421. doi:10.1111/j.1600-0714.1989.tb01575.x 2555479

[10] Jayasooriya PR, Abeyratne S, Ranasinghe AW, Tilakaratne WM Focal epithelial hyperplasia (Heck’s disease): report of two cases with PCR detection of human papillomavirus DNA. Oral Dis. 2004;10(4):240-243. doi:10.1111/j.1601-0825.2004.01012.x 15196147

[11] Bassioukas K, Danielides V, Georgiou I, Photos E, Zagorianakou P, Skevas A Oral focal epithelial hyperplasia. Eur J Dermatol. 2000;10(5):395-397. 10882951

[12] Ozden B, Gunduz K, Gunhan O, Ozden FO A case report of focal epithelial hyperplasia (Heck’s disease) with PCR detection of human papillomavirus. J Maxillofac Oral Surg. 2011;10(4):357-360. doi:10.1007/s12663-011-0184-2 23204755 PMC3267916

[13] Archard HO, Heck JW, Stanley HR Focal epithelial hyperplasia: an unusual oral mucosal lesion found in Indian children. Oral Surg Oral Med Oral Pathol. 1965;20:201-212. doi:10.1016/0030-4220(65)90192-1 14322615

[14] González LV, Gaviria AM, Sanclemente G, Clinical, histopathological and virological findings in patients with focal epithelial hyperplasia from Colombia. Int J Dermatol. 2005;44(4):274-279. doi:10.1111/j.1365-4632.2005.02321.x 15811076

[15] Wu JSA, Florian MC, Rodrigues DA, Tomimori J Skin diseases in indigenous population: retrospective epidemiological study at Xingu Indigenous Park (XIP) and review of the literature. Int J Dermatol. 2017;56(12):1414-1420. doi:10.1111/ijd.13716 28791692

[16] Eversole LR Clinical Outline of Oral Pathology: Diagnosis and Treatment. People’s Medical Publishing House; 2011.

[17] Australian Institute of Health and Welfare Australia’s Health, 2018. Australian Institute of Health and Welfare; 2018.

[18] Cancer Australia Aboriginal and Torres Strait Islander Cancer Statistics. Australian Government; 2018.

[19] Banham D, Roder D, Keefe D, ; CanDAD Aboriginal Community Reference Group and other CanDAD investigators Disparities in cancer stage at diagnosis and survival of Aboriginal and non–Aboriginal South Australians. Cancer Epidemiol. 2017;48:131-139. doi:10.1016/j.canep.2017.04.013 28511150

[20] Australian Institute of Health and Welfare Cancer in Aboriginal and Torres Strait Islander people of Australia. Australian Institute of Health and Welfare; 2018.

[21] Brotherton JM, Winch KL, Chappell G, HPV vaccination coverage and course completion rates for Indigenous Australian adolescents, 2015. Med J Aust. 2019;211(1):31-36. doi:10.5694/mja2.50221 31179546

[22] Garland SM, Brotherton JM, Condon JR, ; WHINURS study group Human papillomavirus prevalence among Indigenous and non-Indigenous Australian women prior to a national HPV vaccination program. BMC Med. 2011;9:104. doi:10.1186/1741-7015-9-104 21910918 PMC3182900

[23] Wood ZC, Bain CJ, Smith DD, Whiteman DC, Antonsson A Oral human papillomavirus infection incidence and clearance: a systematic review of the literature. J Gen Virol. 2017;98(4):519-526. doi:10.1099/jgv.0.000727 28150575

[24] Antonsson A, Cornford M, Perry S, Davis M, Dunne MP, Whiteman DC Prevalence and risk factors for oral HPV infection in young Australians. PLoS One. 2014;9(3):e91761. doi:10.1371/journal.pone.0091761 24637512 PMC3956721

[25] Jamieson L, Garvey G, Hedges J, Human papillomavirus and oropharyngeal cancer among Indigenous Australians: protocol for a prevalence study of oral-related human papillomavirus and cost-effectiveness of prevention. JMIR Res Protoc. 2018;7(6):e10503. doi:10.2196/10503 29884604 PMC6015268

[26] Australian Institute of Health and Welfare Housing Circumstances of Indigenous Households: Tenure and Overcrowding. Australian Institute of Health and Welfare; 2014.

[27] de Roda Husman AM, Walboomers JMM, Hopman E, HPV prevalence in cytomorphologically normal cervical scrapes of pregnant women as determined by PCR: the age-related pattern. J Med Virol. 1995;46(2):97-102. doi:10.1002/jmv.1890460203 7636509

[28] Manos MM, Ting Y, Wright DK, Lewis AJ, Broker TR The use of polymerase chain reaction amplification for the detection of genital human papillomaviruses. Cancer Cell. 1989;7:209-214.

[29] García-Corona C, Vega-Memije E, Mosqueda-Taylor A, Association of HLA-DR4 (DRB1*0404) with human papillomavirus infection in patients with focal epithelial hyperplasia. Arch Dermatol. 2004;140(10):1227-1231. doi:10.1001/archderm.140.10.1227 15492185

[30] National Center for Biotechnology Information BLAST: basic local alignment search tool. Accessed April 17, 2020. https://blast.ncbi.nlm.nih.gov/Blast.cgi

[31] Rissel C, Badcock PB, Smith AM, Heterosexual experience and recent heterosexual encounters among Australian adults: the Second Australian Study of Health and Relationships. Sex Health. 2014;11(5):416-426. doi:10.1071/SH14105 25376995

[32] Wierzbicka M, Berkhof JH, Dikkers FG Prophylactic human papilloma virus vaccination in head and neck: indications and future perspectives. Curr Opin Otolaryngol Head Neck Surg. 2019;27(2):85-90. doi:10.1097/MOO.0000000000000525 30694913

[33] Mazza D, Petrovic K, Chakraborty S HPV vaccination of adult women: an audit of Australian general practitioners. Aust N Z J Obstet Gynaecol. 2012;52(6):528-533. doi:10.1111/ajo.12002 23046059

[34] Bennett LK, Hinshaw M Heck’s disease: diagnosis and susceptibility. Pediatr Dermatol. 2009;26(1):87-89. doi:10.1111/j.1525-1470.2008.00830.x 19250415

[35] Syrjänen S Oral manifestations of human papillomavirus infections. Eur J Oral Sci. 2018;126(suppl 1):49-66. doi:10.1111/eos.12538 30178562 PMC6174935

[36] Kreimer AR, Pierce Campbell CM, Lin HY, Incidence and clearance of oral human papillomavirus infection in men: the HIM cohort study. Lancet. 2013;382(9895):877-887. doi:10.1016/S0140-6736(13)60809-0 23827089 PMC3904652

[37] Rounds T, Harvey J Enrollment challenges: recruiting men to weight loss interventions. Am J Mens Health. 2019;13(1):1557988319832120. doi:10.1177/1557988319832120 30789079 PMC6440040

[38] Buston K Recruiting, retaining and engaging men in social interventions: lessons for implementation focusing on a prison-based parenting intervention for young incarcerated fathers. Child Care Pract. 2018;24(2):164-180. doi:10.1080/13575279.2017.1420034 29503596 PMC5815301

[39] Isaacs A, Pepper H, Pyett P, Gruis H, Waples-Crowe P, Oakley Browne M What you do is important but how you do it is more important. Qual Res J. 2011;11:51-61. doi:10.3316/QRJ1101051

